# Progress in Fevers

**Published:** 1903-01-17

**Authors:** 


### Progress in Fevers.

**Typhoid Fever.**—A. Crombie [1] publishes a second series of figures showing the effect of anti-typhoid inoculation on the incidence of the fever in a number of officers invalided from South Africa. The first series indicated that inoculation conferred a certain protection for about six months, after which period the non-inoculated appeared to suffer less. Acting on the suggestion of Professor Wright, who thought that probably the inoculated would prove to be younger and therefore more susceptible men, a special study with regard to age incidence has been made. Of the 187 cases dealt with 85 were not inoculated, 89 were inoculated once, and 13 were inoculated twice. The incidence among the twice-inoculated was just double that among the once-inoculated, and Crombie thinks the double inoculation may be excessive, diminishing the resisting power of the blood to infection. Of the inoculated 60 per cent. were under 26 years of age, against 22 per cent. among the non-inoculated. The incidence per cent. at this age was 63·4 for the non-inoculated, 27·8 for the once-inoculated, and 71·4 for the twice-inoculated. Above the age of 35 years 28 per cent. were not inoculated and 9 per cent. were inoculated, and the incidences per cent. were 12·5 for the non-inoculated, 37·5 for the once-inoculated and 100 per cent. for the twice-inoculated (only one case). The figures show that for inoculated as for non-inoculated the period of greatest susceptibility is from 20 to 25 years, that up to the age of 30 the advantage of one inoculation is distinct, 27 per cent. as against 51 per cent., but that after 30 the position is reversed, and the advantage is with the non-inoculated rather than with the inoculated, the percentage incidences being 14·3 and 27·4 respectively. The figures seem to teach that, although one inoculation gives a decided advantage to the young soldier, the practice is to be avoided at an age when natural susceptibility to the disease is passing away.

A. E. Wright [2] has prepared a comprehensive table showing the incidence, mortality, and case mortality of typhoid fever, actual and also worked out as percentages, among a large number of inoculated and uninoculated persons. The authority for the statistics of each group is mentioned, reference is made to any literature already published concerning each group, and details are given as to place of exposure, duration of observations, and interval elapsing between inoculation and exposure. In a second elaborate table the statistics given in the first are minutely and critically examined with a view to testing their source and trustworthiness, and the presence of any data likely to vitiate them. The cases comprise 24 groups, 18 of which refer to outbreaks during the recent war in South Africa, two refer to outbreaks in the Maidstone and Dublin Asylums, one deals with the British garrisons in Egypt and Cyprus during 1900, and the remaining three deal with outbreaks among large bodies of men in India Professor Wright points out that owing to the differences in exposure to infection, also to the variation in the proportion of inoculated to uninoculated in each group, no figures can be worked out showing the average diminution in incidence and death-rate of typhoid fever among the inoculated, but, studying each group separately, it can be seen that with three exceptions, viz., the City Imperial Volunteers, and the staffs of the Portland Hospital and of the Imperial Yeomanry Branch Hospital at Pretoria, there was at least a twofold reduction in cases of typhoid fever among the inoculated, while in some cases a still greater reduction in incidence was achieved, varying from six-fold to twenty-eight-fold. Approximately, too, the case mortality was reduced one half in the inoculated. Taking the combined effect of the diminished incidence and diminished case mortality the reduction in death rate among the inoculated is seldom less than a four-fold reduction and frequently exceeds it. The minimum reduction chronicled is two-fold in the case of the City Imperial Volunteers. Professor Wright strongly emphasises the point that in protective inoculation some risk is incurred where the resistance of the patient is low, naturally, or from a previous attack of typhoid fever; or where inoculation is made amidst infected surroundings; or where an excessive dose is given, or reinoculation is too soon resorted to. The last named practices may leave the system less instead of better protected from attack, and taken in conjunction with the smallness of his figures, this fact may explain the adverse conclusions of Crombie mentioned above.

With regard to the prevention of typhoid, what is described as "the tea-kettle policy," [3] that is the boiling of all water used for drinking purposes, although highly necessary, is not to be the only measure relied on. In New York City, in one week of last August, over two hundred cases of typhoid fever were notified, although repeated examination of the water supply failed to find any evidence of typhoid contamination. In Chicago the disease is most prevalent in districts where the water supply is least bad, but where much raw vegetable obtained from market gardens flooded with the overflow from cesspools is consumed. The Health Commissioner of Chicago recommends, in addition to boiling drinking water, washing all raw vegetables in boiled water and protecting food from flies. Sulphate of copper, one pound to two and a half gallons of water is the best and cheapest disinfectant for stools and urine. C. Childs[4] refers to the abstract report of the Commission appointed to investigate the extensive outbreak of typhoid fever in the United States Volunteer Camps in 1898. The finding of the Commission is notable. They bring forward strong and very carefully ascertained evidence in support of the conclusion that "infected water was not the great factor in the causation of the disease." After the suspected water had been replaced by a water of proved purity the epidemics in many instances rapidly increased instead of abating. In one case, that of the 7th Army Corps, encamped close to and in Jacksonville, the inhabitants and the volunteers—about 30,000 of each—were supplied with water from the same source. There were only a few sporadic cases of typhoid among the townsfolk at a time when 60 odd cases were being admitted daily to the camp hospitals. The verdict of the Commission is that infection from man to man and from tent to tent were very important factors in the spread of the epidemic. Elsewhere Dr. Childs[5] and others point out that if the contention of Dr. Leigh Canney that water contamination is the sole source of enteric infection be too readily accepted, the eyes of investigators will be closed to other numerous and possible sources of infection. The danger from infected soil, from flies, and from infected excreta must be steadily borne in mind. M. A. Veeder[6] also emphasises the danger of the last-mentioned sources of infection. No slur is cast on the fine work done by the Army Medical Corps[7] when it is urged that the sanitary lessons taught by the British experiences in South Africa and those of the United States Army in the Spanish-American war should be pressed well home to the Secretary of State for War and to his Majesty's other advisers in dealing with Army Medical Reorganisation.

[1] Lancet, Aug. 16. [2] Ibid., Sept. 6. [3] Med. Rec., Sept. 2 [4] Brit. Med. Jour., July 26. [5] Ibid., Nov. 1, p. 1446. [6] Med Rec., July 26. [7] Brit. Med. Jour., Sept. 20, p. 899.

*(To be Continued.)*

